# Matrilin-3 Chondrodysplasia Mutations Cause Attenuated Chondrogenesis, Premature Hypertrophy and Aberrant Response to TGF-β in Chondroprogenitor Cells

**DOI:** 10.3390/ijms150814555

**Published:** 2014-08-21

**Authors:** Chathuraka T. Jayasuriya, Fiona H. Zhou, Ming Pei, Zhengke Wang, Nicholas J. Lemme, Paul Haines, Qian Chen

**Affiliations:** 1 Department of Orthopaedics, Warren Alpert Medical School of Brown University, CORO West, Suite 402A, 1 Hoppin Street, Providence, RI 02903, USA; E-Mails: chathuraka_jayasuriya@brown.edu (C.T.J.); zwang@chartercare.org (Z.W.); nicholas_lemme@brown.edu (N.J.L.); phaines@bu.edu (P.H.); 2 Sansom Institute of Health Research, Department of Pharmacy and Medical Sciences, University of South Australia, Adelaide 5000, Australia; E-Mail: fiona.zhou@unisa.edu.au; 3 Stem Cell and Tissue Engineering Laboratory, Department of Orthopaedics, West Virginia University, Morgantown, WV 26506, USA; E-Mail: mpei@hsc.wvu.edu

**Keywords:** matrilin-3, multiple epiphyseal dysplasia, spondyloepimetaphyseal dysplasia, chondroprogenitor, chondrogenesis, TGF-β

## Abstract

Studies have shown that mutations in the matrilin-3 gene (*MATN3*) are associated with multiple epiphyseal dysplasia (MED) and spondyloepimetaphyseal dysplasia (SEMD). We tested whether *MATN3* mutations affect the differentiation of chondroprogenitor and/or mesenchymal stem cells, which are precursors to chondrocytes. ATDC5 chondroprogenitors stably expressing wild-type (WT) *MATN3* underwent spontaneous chondrogenesis. Expression of chondrogenic markers collagen II and aggrecan was inhibited in chondroprogenitors carrying the MED or SEMD *MATN3* mutations. Hypertrophic marker collagen X remained attenuated in WT *MATN3* chondroprogenitors, whereas its expression was elevated in chondroprogenitors expressing the MED or SEMD mutant *MATN3* gene suggesting that these mutations inhibit chondrogenesis but promote hypertrophy. TGF-β treatment failed to rescue chondrogenesis markers but dramatically increased collagen X mRNA expression in mutant *MATN3* expressing chondroprogenitors. Synovium derived mesenchymal stem cells harboring the SEMD mutation exhibited lower glycosaminoglycan content than those of WT *MATN3* in response to TGF-β. Our results suggest that the properties of progenitor cells harboring *MATN3* chondrodysplasia mutations were altered, as evidenced by attenuated chondrogenesis and premature hypertrophy. TGF-β treatment failed to completely rescue chondrogenesis but instead induced hypertrophy in mutant *MATN3* chondroprogenitors. Our data suggest that chondroprogenitor cells should be considered as a potential target of chondrodysplasia therapy.

## 1. Introduction

Endochondral bone formation is the primary means of skeletal development in vertebrates. During this process, growth plate chondrocytes undergo rapid proliferation and differentiation into mature chondrocytes. These chondrocytes further differentiated into hypertrophic chondrocytes that, in time, ossify as the diaphysis elongates resulting in long bone growth. The crippling effects of chondrodysplasia illustrate the importance of cartilage development to the process of normal bone growth. Multiple epiphyseal dysplasia (MED) [1] and matrilin type spondyloepimetephyseal dysplasia (SEMD, matrilin type) [2] are two forms of chondrodysplasia associated with mutations in the matrilin-3 (*MATN3*) gene. *MATN3* is a small non-collagenous extracellular matrix (ECM) protein consisting of a von Willebrand factor A (vWFA) domain, four consecutive epidermal growth factor (EGF) repeats and a single coiled-coil domain [3]. *MATN3* is specific to cartilage tissue and highly expressed by growth plate chondrocytes during development [3,4]. Despite our current understanding of its molecular interaction with other cartilage ECM proteins, such as type II/IX collagens [5,6], cartilage oligomeric matrix protein COMP [7] and matrilin-1 (MATN1) [3,8,9,10], the biological role of *MATN3* remains largely unknown.

MED is characterized by delayed or irregular epiphyseal ossification often followed by the early onset of osteoarthritis in patients [11,12,13]. Currently there are more than 13 known MED associated autosomal dominant missense mutations have been mapped to *MATN3*’s vWFA protein domain [14,15,16,17,18,19]. A single tryptophan to arginine (R121W) point mutation in the β-strand of the vWFA domain has been identified in approximately one third of all MED patients, making it the most common *MATN3* mutation to cause this disorder [1,17]. Furthermore, an autosomal recessive cysteine to serine (C304S) point mutation within the first EGF-like domain of *MATN3* has been identified in patients with SEMD [2]. 

Studies to analyze the underlying mechanisms of chondrodysplasia have previously focused on the effects of the *MATN3* missense mutations on chondrocytes. *In vitro* studies have been conducted using primary bovine and chicken chondrocytes, which transiently over-expressed MED (R116W) and SEMD (C299S) *MATN3* mutations, the murine analogs of the human R121W and C304S *MATN3* mutations, respectively [16,20]. These mutations led to disturbed protein trafficking to the Golgi and ultimately resulted in cellular retention of MATN3 in the endoplasmic reticulum of cells. These data suggest that cytosolic accumulation of MATN3 protein may be an underlying pathophysiological event responsible for chondrodysplasia [16,20]. Additionally, an *in vivo* study using knock-in mice carrying the murine equivalent of the MED associated *MATN3* point mutation (V194D) has shown that this mutation results in disregulated chondrocyte proliferation, apoptosis, ER stress response and the development of chondrodysplasia [21]. 

While these previous studies helped to establish that MATN3 is an important ECM protein in regulating cartilage development and homeostasis, they did not address whether chondrodysplasia associated *MATN3* mutations can also affect chondroprogenitors, a precursor cell population that gives rise to chondrocytes. Chondroprogenitors reside in the resting zone, peri-chondrium, growth plate groove of Ranvier, articular cartilage and neighboring tissues in the joint (including synovium) [22]. Chondroprogenitors, which derive from mesenchymal stem cells, are crucial for proper endochondral ossification and bone development through chondrogenesis to form chondrocytes upon induction by growth factors such as TGF-β. During this differentiation process, chondrocytes undergo sequential, well-coordinated events including proliferation, synthesis of chondrogenic markers such as collagen II (*Col2a1*) and aggrecan (*Acan*), and eventually, hypertrophy and synthesis of hypertrophic marker collagen X (*Col10a1*). Unlike *MATN1*, which is expressed mainly by post-mitotic mature chondrocytes [23], *MATN3* is predominantly expressed during early chondrogenesis in the growth plate [10]. Thus, *MATN3* mutations may affect not only chondrocytes but also mesenchymal stem cell derived chondroprogenitors that harbor such mutations. Alteration of these precursor cells may affect the microenvironment of the ECM within the growth plate or articular cartilage and the downstream events during chondrocyte differentiation, thereby contributing to the pathogenesis of MED and SEMD. 

To test this hypothesis, we established stable chondroprogenitor cell lines harboring either the wild-type (WT), MED or SEMD mutant *MATN3* gene in ATDC5 cells, which are commonly recognized chondroprogenitor cells for studying chondrogenesis [24,25]. We analyzed the alteration of expression of chondrogenic, as well as hypertrophic, markers in these cell lines. Additionally, we transfected primary porcine synovium derived mesenchymal stem cells (SDMSCs) [26,27] harboring these mutations, which undergo differentiation upon induction with TGF-β in a pellet culture system. Here we show that *MATN3* mutations, especially SEMD *MATN3*, significantly inhibit chondrogenesis of mesenchymal stem cells and chondroprogenitors. Chondroprogenitors harboring *MATN3* mutations undergo premature hypertrophy. TGF-β treatment fails to rescue chondrogenesis but instead promotes hypertrophy in chondroprogenitors harboring *MATN3* mutations.

## 2. Results

### 2.1. Establishment of Stable ATDC5 Cell Lines Harboring MED and SEMD Associated MATN3 Mutations

**Figure 1:**
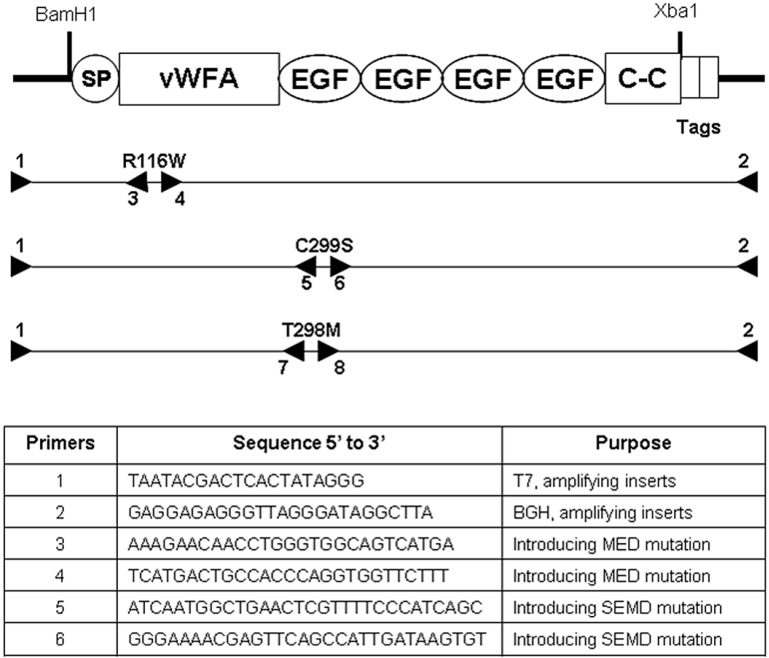
Generating multiple epiphyseal dysplasia (MED) and spondyloepimetaphyseal dysplasia (SEMD) mutant *MATN3* gene constructs. Relative annealing locations of primers used to introduce the MED mutation (R116W) into the vWFA domain, and the SEMD mutation (C299S) into the first EGF-like domain, of the *MATN3* gene. The purpose and sequence of each primer used are listed in the table. SP: signal peptide; C–C: coil–coil domain; Tags: V5 and 6xHis.

**Figure 2:**
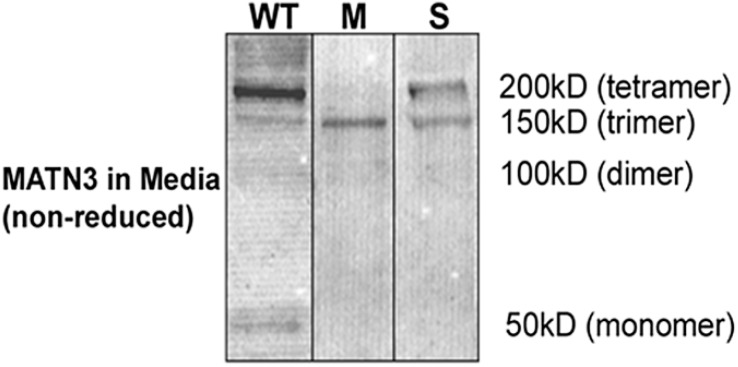
Wild-type (WT) and mutant *MATN3* protein production by stably transfected ATDC5 cell lines. Western blot analysis using an anti-V5 antibody to detect recombinant *MATN3* protein in media of stably transfected with WT, MED or the SEMD *MATN3* gene.

To better understand the function of *MATN3* during chondrogenesis, we stably transfected the ATDC5 murine chondroprogenitor cell line with a WT *MATN3* gene construct or constructs carrying the *MATN3* point mutations associated with either MED or SEMD, respectively (Figure 1). Several cell lines were established by stably transfecting ATDC5 cells with WT, MED or SEMD *MATN3* gene constructs. The full length WT MATN3 protein that was secreted into the cell media formed primarily tetramers along with trimer, dimer and monomer (Figure 2), which is similar to the results previously described in chondrocytes [16,20]. The MED MATN3 protein existed mainly in the form of trimer, while the SEMD MATN3 existed in the form of tetramer and trimer (Figure 2). 

### 2.2. WT MATN3 Induces Spontaneous Chondrogenesis, while MED and SEMD Mutations Diminish MATN3 Induced Chondrogenic Effects, in Chondroprogenitors 

**Figure 3:**
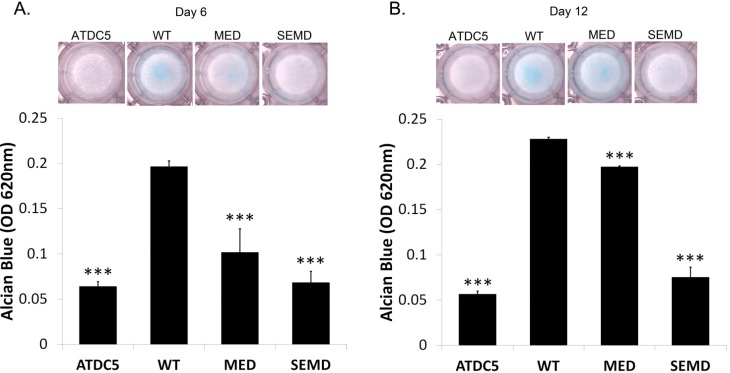
MED and SEMD associated point mutations reduce MATN3 induced chondrogenesis of ATDC5 chondroprogenitors. (**A**) Cells were fixed and stained for proteoglycan accumulation using alcian blue on day 6 and (**B**) day 12. Stains were extracted with 6 M GuHCl and intensities were quantified. *** *p* ≤ 0.005 *vs*. WT *MATN3* stably transfected cells.

**Figure 4:**
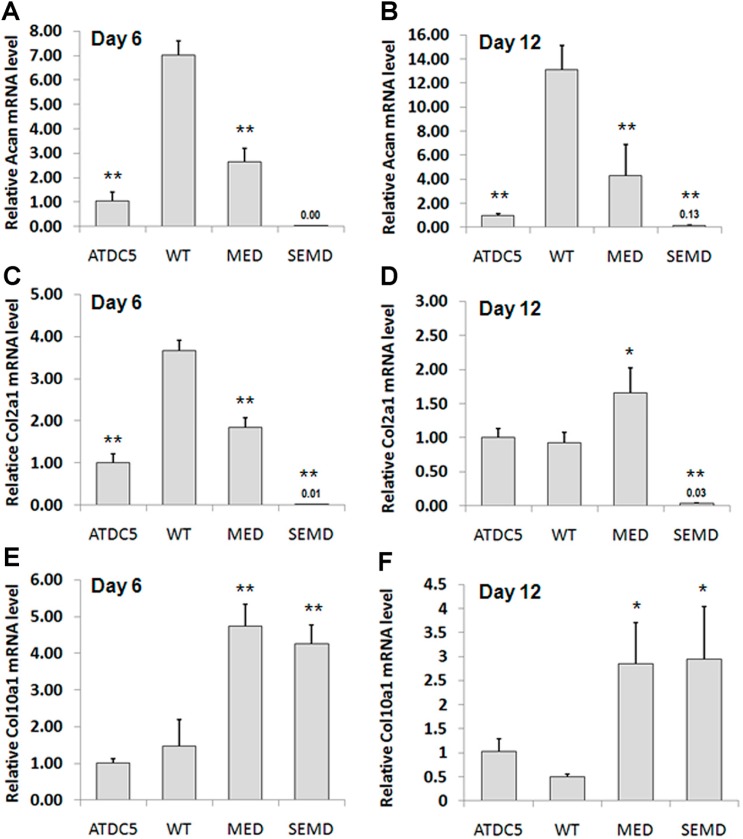
Chondrogenesis and hypertrophic marker expression in WT and mutant *MATN3* transfected ATDC5 chondroprogenitors. (**A**,**B**) Aggrecan mRNA levels; (**C**,**D**) collagen II mRNA levels; (**E**,**F**) collagen X mRNA levels as analyzed by real time RT-PCR in untransfected and *MATN3* transfected ATDC5 cells on days 6 and 12, respectively. * *p* ≤ 0.05 , ** *p* ≤ 0.01 *vs*. WT *MATN3* stably transfected ATDC5 cells.

To determine the extent of chondrogenesis in ATDC5 clones expressing WT or mutant *MATN3* genes, total proteoglycan content was measured by alcian blue staining. After culturing in the absence of chondrogenesis induction medium for 6 and 12 days, ATDC5 cells exhibited no noticeable alcian blue staining (Figure 3A,B). However, positive staining was observed in the cells stably transfected with WT *MATN3*. Cells expressing MED *MATN3* exhibited reduced alcian blue staining relative to those expressing WT *MATN3*. Moreover, SEMD *MATN3* expressing cells exhibited nearly no alcian blue staining on both day 6 and 12 (Figure 3A,B). Real-time RT-PCR analysis demonstrated that WT *MATN3* enhanced *Acan* mRNA levels from 7 fold (day 6) to more than 13 fold (day 12) relative to parental ATDC5 cells (Figure 4A,B). Furthermore, WT *MATN3* expression resulted in a transient up regulation in *Col2a1* mRNA peaking at day 6 and returning to basal level by day 12 (Figure 4C,D). WT *MATN3* transfection did not significantly alter *Col10a1* mRNA expression in ATDC5 cells (Figure 4E,F). In contrast, the MED *MATN3* transfected cells exhibited significant suppression of Acan mRNA levels relative to WT *MATN3* transfected cells, while SEMD *MATN3* transfection abolished *Acan* and *Col2a1* mRNA levels (Figure 4A–D). However, the hypertrophic marker *Col10a1* was only upregulated significantly in ATDC5 cells expressing the *MATN3* mutations at day 6 (Figure 4E), and remained high at day 12 (Figure 4F).

### 2.3. TGF-β1 Is Incapable of Restoring Normal Chondrogenic Phenotype to Cells Expressing the MED or SEMD MATN3 Mutation

**Figure 5:**
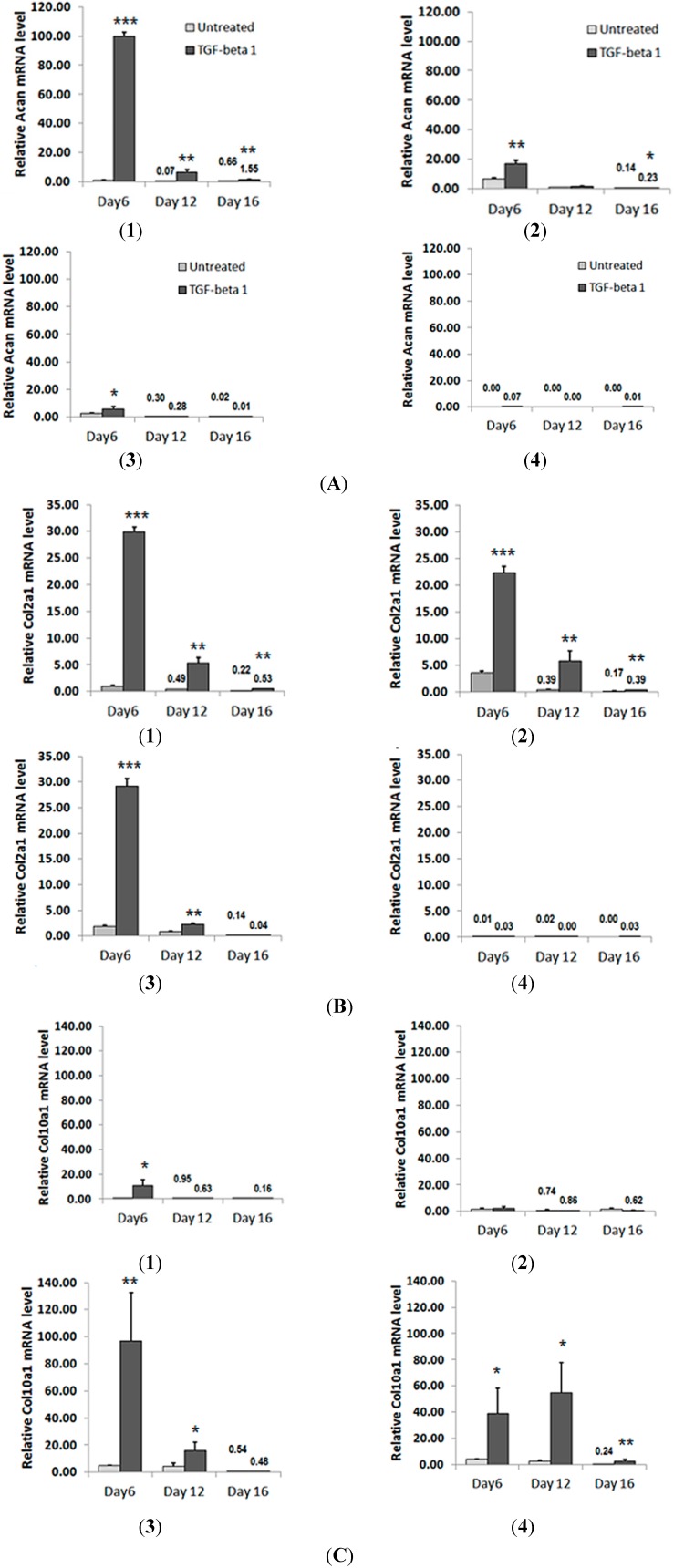
Effects of TGF-β1 on the chondrogenic differentiation of ATDC5 cells expressing WT, MED or SEMD *MATN3* during a 16 day time course. ATDC5 cells were seeded into 12 well culture plates and the growth media replaced with DMEM:F12 supplemented with or without TGF-Β1 (10 ng/mL). On Days 6, 12 and 16, gene expression was quantified in each group: untransfected ATDC5; WT *MATN3*; MED; SEMD, respectively. (**A**(**1**–**4**)) expression of *Acan*; (**B**(**1**–**4**)) *Col2a1* and (**C**(**1**–**4**)) *Col10a1* was measured using real-time RT-PCR. Relative quantities less than 1.00 are so indicated for clarity. * *p* ≤ 0.05, ** *p* ≤ 0.01 , *** *p* ≤ 0.005 *vs.* TGF-β1 untreated groups.

To determine whether TGF-β1 can rescue suppressed chondrogenesis in mutant chondroprogenitors, chondrogenic differentiation in response to TGF-β1 treatment was examined by real-time RT-PCR. TGF-β1 treatment of ATDC5 cells enhanced *Acan* mRNA expression by approximately 100 fold and *Col2a1* by 29 fold on day 6, which remained significantly elevated, with respect to untreated ATDC5 controls, at day 12 and day 16 (Figure 5 A(1),B(1)). Even though WT *MATN3* can induce spontaneous chondrogenesis in chondroprogenitors, TGF-β1 treatment helped to further enhance *Acan* and *Col2a1* expression, however, to a lesser extent than observed in the parental ATDC5 control cells (Figure 5 A(2),B(2)). In MED *MATN3* cells, TGF-β1 treatment increased *Acan* and *Col2a1* at earlier time point (day 6), but was largely ineffective at later time points (Figure 5 A(3),B(3)). In SEMD *MATN3* cells, neither *Acan* nor *Col2a1* levels were increased in response to TGF-β1 stimulation (Figure 5 A(4),B(4)). However, the hypertrophic chondrocyte marker *Col10a1* was greatly induced by TGF-β1 treatment in both MED and SEMD *MATN3* chondroprogenitors, while its levels remained low in WT *MATN3* cells and the parental ATDC5 cells (Figure 5 C(1–4)).

### 2.4. The Effect of WT and Mutant MATN3 on Chondrogenesis Is Consistent among Different Cell Lines

**Figure 6:**
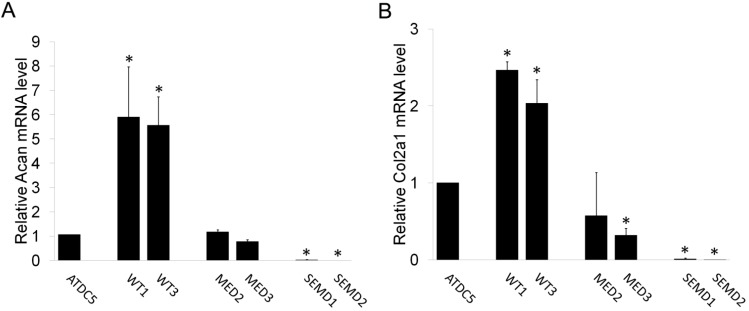
Low and high expression clones of WT and mutant *MATN3* transfected cell lines show similar trend of chondrogenesis marker expression. (**A**) Gene expression of aggrecan and (**B**) type II collagen in low and high expression WT or mutant *MATN3* stably transfected ATDC5 cell lines as analyzed by real time RT-PCR 24 h post seeding. * *p* ≤ 0.05 *vs.* untransfected ATDC5 cells.

We generated multiple stable cell lines harboring each *MATN3* transgene (Table 1). The above data were obtained from WT3, MED2, and SEMD2. To test whether the effect of WT and mutant *MATN3* on chondroprogenitor cells were consistent among different cell lines, we quantified the basal mRNA expression levels of chondrogenesis markers *Acan* and *Col2a1* in two cell lines that expressed lower and higher expression level of each *MATN3* transgene, respectively. Our results showed that the effect of *MATN3* on chondrogenesis largely depended on the type of *MATN3* expressed (*i.e.*, whether it is WT or MED or SEMD), rather than the expression levels of each *MATN3* transgene (Figure 6). 

**Table 1:** Wild-type or mutant *MATN3* gene expression in stably transfected ATDC5 cell lines.

Cell Lines	Generated Clones	*MATN3* Expression Levels (Relative to Parental ATDC5)
WT *MATN3*	WT1	2.6
	WT2	18.4
	WT3	25.7
MED *MATN3*	MED1	1.5
	MED2	1.9
	MED3	17.2
SEMD *MATN3*	SEMD1	6.9
	SEMD2	46.8
	SEMD3	1122

### 2.5. Synovium Derived Mesenchymal Stem Cells Expressing SEMD MATN3 Exhibit Diminished Chondrogenesis Potential

**Figure 7:**
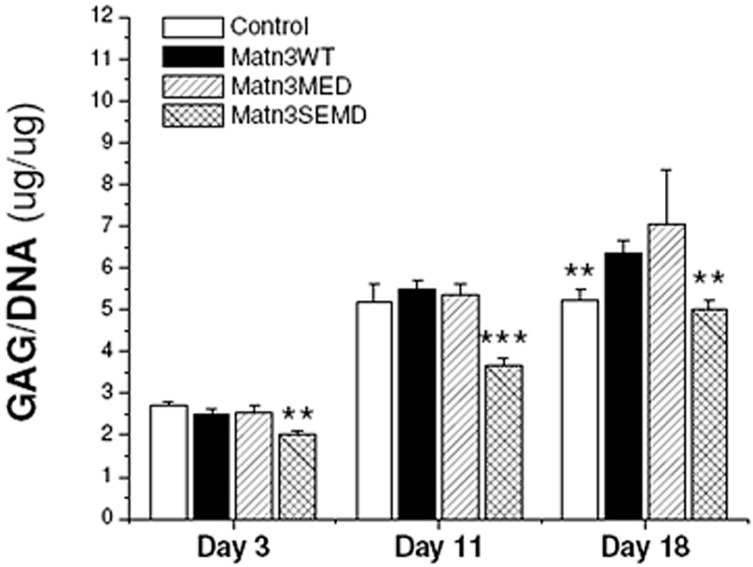
SEMD *MATN3* transfected primary synovium derived mesenchymal stem cells exhibit reduced GAG content. GAG content was measured using a dimethylmethylene blue assay and values were normalized to DNA content. Controls are cells transfected with empty vector. ** *p* ≤ 0.01, *** *p* ≤ 0.005 *vs.* WT *MATN3* transfected cells.

**Figure 8:**
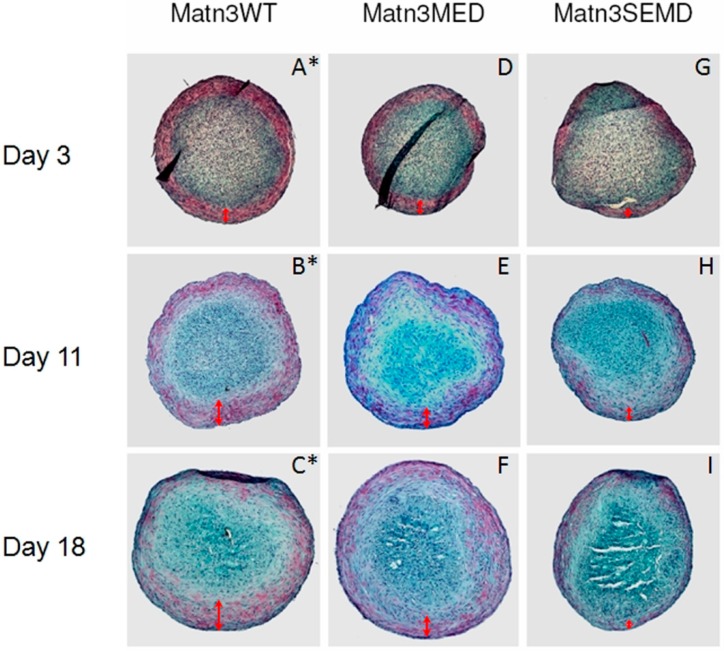
The SEMD *MATN3* mutation hinders *MATN3* induced chondrogenesis of primary porcine synovium derived stem cells. (**A**–**C**) Extent of chondrogenesis of WT *MATN3* transfected; (**D**–**F**) MED *MATN3* transfected and (**G**–**I**) SEMD *MATN3* transfected porcine SDMSC pellets as indicated by Safranin-O staining. Red double headed arrows indicate the depth of each pellet’s peripheral cartilaginous zone that stained positive with Safranin-O. * Note: **A**, **B** and **C** were reference panels from a previous study [26] for the purpose of comparing the extent of chondrogenesis in WT *MATN3* transfected cells *vs.* MED or SEMD *MATN3* transfected cells.

To investigate the effect of WT and mutant *MATN3* gene expression in primary stem cells, we transfected porcine synovium derived mesenchymal stem cells (SDMSCs) with WT, MED or SEMD *MATN3* gene construct respectively. They were then incubated in the presence of TGF-β to induce chondrogenesis in pellet culture. In comparison to the WT *MATN3* expressing SDMSC, the GAG content was significantly lower in the SEMD *MATN3* expressing SDMSC after 3 and 11 days of incubation. The GAG content of the SEMD *MATN3* expressing SDMSC or non-transfected SDMSC was significantly lower than those transfected with WT *MATN3* after 18 days incubation (Figure 7). Histological analysis of the SDMSC pellet culture indicates that the size of the pellet of the SEMD *MATN3* expressing cells was significantly smaller than that of WT *MATN3* or MED *MATN3* expressing cells (Figure 8). Furthermore, the chondrogenesis area as indicated by red Safranin-O staining in the pellet was significantly smaller in the SEMD *MATN3* expressing cells than that of WT *MATN3* or MED *MATN3* expressing cells (Figure 8). This suggests an overall attenuation of chondrogenesis in the SDMSCs transfected with the SEMD mutant *MATN3* gene.

## 3. Experimental Section 

The ATDC5 murine chondroprogenitor cell line was acquired from RIKEN cell bank, Japan. All cell culture reagents including DMEM:F12, fetal bovine serum, trypsin EDTA solution and Anti V5 antibody were purchased from Invitrogen (Carlsbad, CA, USA). Human Transferrin, Selenite, Trypan blue, Alcian Blue and GuHCl was purchased from Sigma-Aldrich (St. Louis, MO, USA) while TGF-β1 was from Peprotech (Rocky Hill, NJ, USA). 

### 3.1. Cloning and Construction of MATN3 cDNAs

The WT *MATN3* gene construct and mutant *MATN3* construct containing a R116W point mutation (MED), were each cloned into expression vectors of pcDNA3.1/V5-His (Invitrogen, Carlsbad, CA, USA) as previously described [10,20]. Using similar methods, we also generated *MATN3* with the C299S point mutation (SEMD) on mouse sequence NM_010770 (Figure 1). Overlapping PCR was performed to create MATN3 cDNA carrying the SEMD mutation using mouse full-length pcDNA3.1-MATN3 and Qiagen hotstart Taq (Valencia, CA, USA). Primer sequences are listed in Figure 1. The size of the 1.4 kb *MATN3* product was confirmed on a 1% agarose gel. The pcDNA plasmid plus the mutated *MATN3* PCR products were digested with BamH1 and Xba1 (Roche, Mannheim, Germany). The SEMD product was then ligated into pcDNA and transformed into competent *E.coli* DH5α cells (Invitrogen, Carlsbad, CA, USA). Colonies grown on ampicillin LB agar were picked and expanded. All plasmids were purified using high pure plasmid isolation kit (Roche, Mannheim, Germany) to check for the *MATN3* inserts using BamH1 and Xba1 digestion. Furthermore, individual SEMD and MED clones were sequenced with T7 and BGH primers and confirmed to contain the desired nucleotide change in *MATN3*. 

### 3.2. Establishing ATDC5 Chondroprogenitor Cell Lines and SDMSCs Harboring MATN3 Mutations

ATDC5 cells were grown in complete media (DMEM:F12, 5% FBS and 10 μg/mL Transferrin, 3 × 10^−8^ M sodium selenite) [24], and maintained at 37 °C and 5% CO_2_. Cells were split into 12 well plates and grown to 95% confluency. Transfection was performed with Lipofectamine 2000 according to manufacture instruction (Invitrogen, Carlsbad, CA, USA). Briefly, 1 μg of DNA was mixed with 2 μL Lipofectamine 2000 in serum free Opti-MEM. Within 20 min at room temperature, the mixture was added to cells. After 24 h, media was changed. G418 (5, 6, 7 or 8 μM) was added in the complete media to select against cells that were not successfully transfected. Untransfected cells were killed by incubation with 4 concentrations of G418 after 4 days. After treatment of 5 μM G4182 for two weeks, several individual clones from each transfected cell line were isolated using sterile loops and cultured to confluency in new 12 well plates with complete media without G418. All ATDC5 clones containing various *MATN3* constructs were subcultured for further gene and protein characterization. Several clones were analyzed for MATN3 protein production/retention (data not shown). For several lines, gene expression analysis was conducted to verify that chondrogenesis marker expression did not greatly vary based on relative *MATN3* expression alone (Figure 6). After this was confirmed, a single clone was selected to represent each WT, MED or SEMD mutant *MATN3* line for experiments. 

CD14 negative isolation was used to acquire primary SDMSCs from porcine synovium, as previously described [26,27,28]. SDMSCs were plated at a cell density of 1.8 × 10^6^ cells per 25 cm culture flask. After 24 h, cells were stably transfected using 8 μg of the previously described WT, MED or SEMD murine *MATN3* constructs and 12 μg of FuGENE 6 (Roche, Indianapolis, IN, USA) transfection mediator, according to the manufacturer’s instruction. 

### 3.3. SDS-Polyacrylamide Gel Electrophoresis and Western Blot Analysis

Stably transfected ATDC5 cells containing the WT or mutant *MATN3* genes as well as the untransfected ATDC5 control cells were grown in monolayer in 6 well culture plates in complete media. After 3 days, cell lysates and supernatants were separately collected for western blot analysis. To concentrate MATN3 proteins from media, 50 μL Ni-Agarose beads (Invitrogen, Carlsbad, CA, USA) were used to isolate His tagged recombinant MATN3 proteins from 1.0 mL of cell supernatants. After 48 h at 4 °C on a shaker, the Ni-agarose beads from all samples were centrifuged and washed 7 times with buffer containing 20 mM potassium phosphate, 500 mM potassium chloride, 10 mM imidazole pH 7.5. Before protein samples were loaded on to 10% SDS PAGE gel, His tagged proteins were dissociated from Ni-Agarose beads by boiling at 95 °C for 10 min in 30 μL 2× SDS gel loading buffer, then put on ice and centrifuged to separate Ni-Agarose beads. For western blot, proteins were transferred onto Immobilon-PVDF membrane (Millipore Corp., Bedford, MA, USA) in 25 mM Tris, 192 mM glycine, and 15% methanol. The membranes were blocked in Odyssey^®^ Blocking Buffer for 1 h and then probed with a mAb against murine V5 (1:5000) (Invitrogen, Carlsbad, CA, USA). IRDye^®^ 800CW Conjugated anti-Mouse (diluted 1:10,000) (LI-COR Biosciences, Lincoln, NE, USA) was used as secondary antibody. The mutant and WT MATN3 with V5 tag proteins were visualized in the 800 nm channel using Odyssey infrared image system according to manufacturer’s instruction (LI-COR Biosciences, Lincoln, NE, USA). Molecular protein marker used is specific for the Odyssey system and is visualized in the 700 nm channel. 

### 3.4. Experimental Cell Culture Conditions

All experiments involving ATDC5 cells were conducted in monolayer cell culture. Each selected WT, MED and SEMD *MATN3* ATDC5 clone and untransfected ATDC5 control were plated in triplicate at 5 × 10^4^ cells/well in 12 well plates in DMEM:F12, 5% FBS and 10 μg/mL Transferrin, 3 × 10^–8^ M sodium selenite). Experiments involving primary SDMSCs were conducted using a previously described pellet culture system [26]. Transfected and mock-transfected (control) primary SDMSCs were detached using trypsin EDTA. 3.0 × 10^5^ cells were centrifuged for 10 min at 500× *g* to obtain cell pellets which were then cultured in 24-well plates for 3, 11 and 18 days in TGF-β1 (10 ng/mL) supplemented High-Glucose DMEM media containing: proline (40 mg/mL), 100 mM dexamethasone, 0.1 mM ascorbic acid 2-phosphate, penicillin (100 U/mL), streptomycin (100 mg/L), insulin (6.25 mg/mL), transferring (6.25 mg/mL), selenous acid (6.25 mg/mL), linoleic acid (5.35 mg/mL) and BSA (1.25 mg/mL). 

### 3.5. RNA Isolation and Real-Time RT-PCR 

Three-hundred microliters of Lysis/binding buffer (Ambion, Austin, TX, USA) was added to each monolayer cell culture well. All samples were collected and immediately processed to isolate total RNA using filter cartridge and buffers from RNAqueous Kit (Ambion, Austin, TX, USA) according to manufacturer’s instructions. Total RNA was quantified using a NanoDrop 2000c (Thermo Scientific, Wilmington, DE, USA), and 500 ng of RNA was used for cDNA synthesis using iScript Reverse Transcription Supermix (Bio-Rad Hercules, CA, USA) for RT-PCR according the manufacturer’s instructions. Quantification of mRNA was performed by RT-PCR with QuantiTect SYBR Green PCR Kit (Qiagen, Valencia, CA, USA) using the synthesized cDNA and primers described in Table 2 via Bio-Rad 96 well thermo cycler. Ribosomal RNA 18S was used as the internal control. The cycle threshold (*C*t) values for specific target gene and 18S of each cDNA test sample (test) and control (ctl) were measured and obtained using Opticon Monitor Analysis Software: Version 2.02 (Bio-Rad, Hercules, CA, USA). In accordance with the delta delta *C*t *(∆∆C*t) method, relative transcript levels were calculated as *x* = 2^(−ΔΔ*Ct*)^, where ΔΔ*C*t = Δ*E* − Δ*C*, Δ*E* = *C*t_test_ − *C*t_18S_; Δ*C* = *C*t_ctl_ − *Ct*_18S_.

**Table 2:** Forward and reverse primer sequences used in gene expression analysis.

Gene	Forward	Reverse
*Acan*	CAGTGCGATGCAGGCTGGCT	CCTCCGGCACTCGTTGGCTG
*Col2a1*	CACACTGGTAAGTGGGGCAAGACCG	GGATTGTGTTGTTTCAGGGTTCGGG
*Col10a1*	GCCAGGAAAGCTGCCCCACG	GAGGTCCGGTTGGGCCTGGT
*MATN3*	TTCCACCCGCGCGCCATATTC	CGTGTCTGTGGCCCCGATGTC

### 3.6. Alcian Blue Staining and Quantification

On Days 6 and 12, media was removed from each of the stable transfected ATDC5 clones and gently washed with sterile 1× PBS to remove detached/dead cells. Cells were fixed in cold 100% methanol for 2 min at −20 °C and gently washed with 1× PBS. Samples were stained overnight with 500 μL of 0.1% Alcian blue in 0.1 M HCl pH = 1.0, plates were washed 3 times in milli-Q water (Millipore, Billerica, MA, USA). Images of Alcian Blue staining were acquired using a Leica MZ6 Stereo microscope (Leica Microsystems, Buffalo Grove, IL, USA). Blue stains were extracted for quantification using 300 μL of 6 M GuHCl, which was incubated overnight. Extracted color was measured in a 96 well plate at 620 nm using a spectrophotometer. 

### 3.7. Histology and Safranin-O Staining of Primary SDMSC Cell Pellets

Primary SDMSC cell pellets were fixed using 4% paraformaldehyde in PBS (pH 7.4) for 24 h at 4 °C followed by dehydration in ethanol. Specimens were then cleared using xylene and embedded in paraffin. Individual sections (5 μm) were then cut and subsequently stained with safranin-O/fast green (for visualization of GAG) and counterstained with hematoxylin [26]. 

### 3.8. GAG Quantification with Dimethylmethylene Blue Assay

After 3, 11 and 18 days in culture, primary SDMSC cell pellets were digested in buffer containing papain (125 mg/mL), 100 mM phosphate, 10 mM EDTA and 10 mM cysteine. Using the digested lysates, a dimethylmethylene blue assay [29] was conducted to measure GAG content with a spectrophotometer (PerkinElmer, Norwalk, CT, USA). Bovine chondrotin sulfate was used to generate the standard curve utilized for GAG quantification. DNA content, as measured by Hoechst 33258 dye and a spectrofluorometer (QM-1; Photon Technology International, South Brunswick, NJ, USA), was used to control for cell number. Type I calf thymus DNA (Sigma, St. Louis, MO, USA) was used to generate the standard curve utilized for DNA quantification. 

### 3.9. Statistical Analysis

All quantitative data are expressed as mean ± SDM (standard deviation of the mean). Statistics were conducted using one-way ANOVA followed by *post-hoc* analysis. In Figure 5, a Student’s *t*-test was used to compare TGF-β1 treated and untreated groups. Differences between groups were considered statistically significant when *p*-value ≤0.05.

## 4. Discussion 

Extracellular matrix (ECM) proteins have been proposed to provide a microenvironment suitable for maintaining the chondrogenic phenotype of differentiating mesenchymal stem cells [26], which is required for proper musculoskeletal development. However, it is not clear whether mutations of an ECM gene can affect chondroprogenitor cell differentiation into chondrocytes. Several chondrodysplasia mutations including MED and SEMD have been mapped to *MATN3*, a gene encoding a non-collagenous matrix glycoprotein [2,13,14,16,17,20,30]. Previous studies have utilized transient transfection of the MED (R116W) and SEMD (C299S) mutant *MATN3* gene constructs into chondrocytes to understand their effects [16,20]. Until now, it has been generally assumed that the molecular defects of MED and SEMD reside in chondrocytes, which were thought to be the only type of cells within cartilage. However, in recent years, it has been shown that cartilage contains a population of progenitor cells of the mesenchymal origin that can differentiate along the chondrogenic lineage, which is vital for cartilage development [31,32].

The main goal of our study was to understand the role of the WT *MATN3* gene, as well as MED and SEMD mutant *MATN3* genes, during the process of chondroprogenitor cell differentiation, which is a crucial preamble to articular cartilage tissue formation and endochondral ossification during skeletal development. To do this, we established several chondroprogenitor cell lines by stably transfecting ATDC5 cells with the wild type, MED or SEMD *MATN3* gene constructs. Using these lines, we demonstrated that chondroprogenitors expressing the WT *MATN3* gene show signs of spontaneous chondrogenic differentiation. Relative to the WT *MATN3* transfected cells, ATDC5 cells carrying the MED *MATN3* mutation exhibited reduced chondrogenic marker expression at most tested time points. This diminishment was even more pronounced in cells carrying the SEMD *MATN3* mutation. Consistent with mRNA analysis of chondrogenesis markers, alcian blue staining intensities indicated a reduction of proteoglycan accumulation in ATDC5 cells carrying either the MED or SEMD *MATN3* mutant construct. The SEMD *MATN3* group exhibited the greatest reduction in alcian blue staining intensity relative to wild-type *MATN3* expressing cells. Similarly, primary SDMSCs expressing the SEMD *MATN3* mutant construct exhibited a significant reduction of GAG content compared to the WT *MATN3* group. In particular, our experiments with SDMSCs demonstrated that the presence of the SEMD associated mutant *MATN3* gene can hinder the process of chondrogenesis in primary cells, in a similar way to that observed in the ATDC5 cell line, confirming that this phenomenon is not simply an isolated event observed in one particular cell model, but rather a definitive abnormality observed in cells that express this *MATN3* mutation. 

Furthermore, both the MED *MATN3* and SEMD *MATN3* expressing ATDC5 cells exhibit elevated type X collagen mRNA levels relative to the control cell group whereas WT *MATN3* cells remain at normal levels, strongly suggesting that the MED and SEMD associated *MATN3* mutations induce premature hypertrophy during chondroprogenitor cell differentiation. Taken together, these findings suggested that the MED and SEMD associated *MATN3* mutation can dramatically impact the early stages of chondrogenesis in both chondroprogenitor cells by abrogating proteoglycan accumulation. This is the first study to report the evidence suggesting that the MED and SEMD mutations, and the SEMD mutation in particular, affect early chondrogenesis of progenitor cells. Chondrogenesis, as part of the endochondral ossification process, is a crucial step that can influence skeletal formation [33,34]. Interestingly, the significant elevation of the hypertrophic markers observed in the MED and SEMD *MATN3* expressing cells also suggest that these *MATN3* mutations may promote premature hypertrophy. Because the coordination between the proliferative and hypertrophic stage of chondrogenesis is critical to the longitudinal bone growth during skeletal development [35], the occurrence of premature hypertrophy may shorten the chondrogenic process thereby negatively impacting bone growth [36]. 

Previous studies have demonstrated that TGF-β1 treatment can promote chondrogenesis while also preventing chondrocyte hypertrophy [37,38]. To determine whether TGF-β1 can remedy the reduced chondrogenic potential and increased hypertrophy that is typical of mutant *MATN3* expressing progenitor lines, we treated these cells with TGF-β1 for up to 16 days in culture. TGF-β1 treatment appeared to enhance aggrecan expression in normal chondroprogenitor cells as well as WT-*MATN3* transfected cells but failed to elevate its expression in the cells harboring either MED or SEMD *MATN3* mutations. The inability of TGF-β1 to rescue the attenuation of the chondrogenic marker aggrecan in the MED and SEMD expressing cells suggests that TGF-β1 is not effective in restoring the normal chondrogenic phenotype in mutant chondroprogenitor cells. What is more, TGF-β1 treatment further increased the already elevated type X collagen mRNA levels in both MED and SEMD *MATN3* expressing cells. This suggests that the combined effect of TGF-β1 and mutant MATN3 proteins may expedite terminal differentiation in these chondroprogenitor cells.

Overall, the present study presents evidence supporting the hypothesis that cartilage ECM protein *MATN3* provides a microenvironment suitable for promoting chondrogenic differentiation and inhibiting hypertrophy for chondroprogenitors. We demonstrate here for the first time that MED or SEMD chondrodysplasia associated *MATN3* missense mutations have a dominant effect to render attenuated chondrogenesis and premature hypertrophy in chondroprogenitor cells. 
